# Moral Agreement With Punished Acts Decreases Perceptions of Punisher Legitimacy and Willingness to Obey the Law

**DOI:** 10.1177/01461672251385772

**Published:** 2025-11-20

**Authors:** Raihan Alam, Tage S. Rai

**Affiliations:** 1 University of California, San Diego, USA

**Keywords:** morality, punishment, procedural justice, compliance

## Abstract

Punishment is a critical mechanism through which society regulates behavior, yet its efficacy depends on how observers interpret the legitimacy of punishers. Across five experiments, we examine how moral agreement with punished acts shapes perceptions of punishers’ legitimacy and willingness to obey laws. Experiment 1 finds that when observers morally agree with punished acts, they perceive punishers as less legitimate and report lower willingness to obey laws. Experiment 2 shows that this effect extends to compliance with a new, specific law introduced by the punishing authority. Experiment 3 finds that when moral agreement and procedural justice are manipulated simultaneously, only moral agreement predicts willingness to obey laws. Experiment 4 replicates these effects among participants with criminal records. Experiment 5 shows that these patterns persist when addressing potential confounds and when moral preferences are weaker. Our findings challenge procedural justice models, highlighting the importance of addressing moral disagreement in policy contexts.

## Introduction

Punishment is one of the primary mechanisms through which societies attempt to regulate behavior and prevent crime. According to rational actor models of crime (Becker, 1968), the threat and actual punishment of criminal behavior deter offenders and the public from engaging in that particular transgression or breaking other laws in the future, a phenomenon known as general deterrence (Apel & Nagin, 2011; Nagin, 2013). On this view, criminal behavior is the product of a cost–benefit analysis wherein offenders engage in crime when its expected utility is positive, and as a result, policies that increase the severity of punishment should deter criminal behavior. However, decades of criminological research suggest that such punitive policies, including mandatory and minimum sentencing laws, three-strike-laws, and even capital punishment, have inconsistent, weak, and sometimes counterproductive effects on crime (Kovandzic et al., 2004; Marvell & Moody, 2001; National Research Council, 2012), raising questions both about the limitations of rational actor models of crime and the conditions under which punishment may successfully deter crime and promote legal compliance.

In contrast to rational actor models which assume that punishment discourages crime by imposing material costs on offenders (Becker, 1968), psychologists have emphasized the importance of procedural justice in fostering perceptions of legitimacy that support compliance with the law. According to procedural justice theory, individuals are most likely to comply with laws and accept legal decisions when they perceive administering authorities as possessing the legitimate right to act on behalf of a collective (Tyler, 2006). Legitimacy is crucial for institutions because it fosters voluntary compliance with rules and authorities even in the absence of punishment or rewards (Tyler, 1997). From this perspective, crime and noncompliance with the law arise when the perceived legitimacy of institutions declines. Correlational and experimental research support this account, demonstrating that perceiving institutions as legitimate predicts greater adherence with the law, reduced criminal behavior, and lower rates of recidivism (Alam & Rai, 2025a; Nivette & Eisner, 2013; Paternoster et al., 1997; Peyton et al., 2019; Roth, 2024; Sunshine & Tyler, 2003; Tyler et al., 2010; Tyler & Jackson, 2013). These relationships emerge cross-culturally (Akdogan et al., 2024; Dawson, 2017; Tankebe et al., 2016), and many legal scholars and policymakers’ view legitimacy-based approaches to compliance as a cornerstone of effective crime control (Tyler, 2023). Crucially, within this literature, scholars assume legitimacy stems from procedural justice, which requires the absence of corruption, the presence of transparency in decision-making, and opportunities to express one’s views, which collectively signal to individuals that authorities behave in a fair, honest, and trustworthy manner (Tyler & Lind, 2001).

At the same time, moral psychology researchers have identified a crucial boundary condition of procedural justice, arguing that its effects on legitimacy are limited when people hold moral convictions about the underlying issues of legal decisions. Moral convictions are deep-seated beliefs that an issue is fundamentally moral or immoral, and which are experienced by individuals as absolute and nonnegotiable (Skitka et al., 2005). When legal decisions conflict with someone’s moral convictions, they may reject legal authorities as illegitimate, regardless of the procedural fairness of the decision-making process (Bauman & Skitka, 2009; Skitka et al., 2009; Skitka & Houston, 2001; Skitka & Mullen, 2002; Mullen & Skitka, 2006). For example, Bauman and Skitka (2009) conducted a national survey experiment on abortion, manipulating both outcomes (having participants imagine whether the U.S. Supreme Court ruled to uphold or overturn legal abortion) and voice—a key antecedent of procedural justice. Voice refers to opportunities individuals are afforded by institutions to express their views in a decision-making process and is a widely accepted manipulation of procedural justice (Brockner et al., 2001; Nivette et al., 2024; Van den Bos, 1999). Their findings showed that while voice typically enhances perceptions of legitimacy, this effect emerged only when moral convictions about abortion were low. However, when moral convictions were high, legitimacy judgments were primarily determined by whether the Supreme Court’s decision aligned with the individual’s moral stance, rather than by procedural factors, such as voice. Other research has found that even when people initially deem the legal process as legitimate, they revise their evaluations when the final decision of legal authorities conflict with moral views they feel strongly about (Mullen & Skitka, 2006; Skitka & Mullen, 2002; Skitka et al., 2009). These relationships have also been documented by researchers in the context of the Supreme Court’s decision to uphold the legalization of physician-assisted suicide in Oregon (Skitka et al., 2009), the Supreme Court’s overturning of *Roe vs. Wade* by the Dobbs decision (Gibson, 2024), and in qualitative analyses of news editorials on the Rodney King case, which showed that concerns about procedural justice and racism were raised more frequently after the officers’ acquittal rather than before (Mullen & Skitka, 2006).

## The Current Research

While prior work has examined how moral convictions shape the acceptance of specific legal and political decisions, much less attention in the literature has been given to their role in generalizing from acceptance of specific laws to shaping broader compliance with other laws enforced by punishing authorities. From a procedural justice perspective, people are most likely to comply with the broader set of laws set forth by legal authorities when those authorities have a reputation for acting in procedurally just ways, as this is most directly relevant for inferring their legitimacy. However, the moral conviction literature suggests that if observers morally agree with one punished act, they may no longer perceive authorities as legitimate regardless of whether authorities enforced the law through just procedures, and as a result, they may be less likely to comply with laws more broadly. In this paper, we tested whether moral agreement with punished acts generalizes to affect subsequent compliance with laws enforced by punishing authorities both broadly and in relation to specific laws, and whether this relationship is mediated by changes in perceived punisher legitimacy. We also investigated whether moral agreement has a stronger effect on perceptions of legitimacy and compliance than procedural justice factors traditionally assumed by researchers to underpin legitimacy. Finally, we examined whether the influence of moral beliefs on compliance is limited to cases involving strong moral attitudes, or whether such effects extend even to issues where individuals have weaker moral preferences. If moral agreement affects legitimacy and broad compliance, and if these effects are stronger than an authority’s reputation for procedural justice even when moral preferences are weak, this would have major implications for procedural justice theory and crime-control strategies; it would suggest that procedural justice approaches to compliance may be more limited than previously assumed in the literature, and that in moralized contexts, alternative mechanisms for compliance warrant further exploration by researchers.

In Experiment 1, which was preregistered, we manipulated moral agreement with a perpetrator’s transgression to test its effect on punisher legitimacy and broader compliance with punishing authorities. We predicted that when participants are exposed to punishment of an act that they morally agree with, they would be less likely to view the punishing body as legitimate and report lower willingness to obey laws in the future. Because we randomly assigned participants to conditions, we can assume that participants’ baseline levels of compliance are equivalent across conditions, and so any difference between conditions in their reported willingness to follow laws results from observing punishment of a specific act that is either morally agreeable or disagreeable. In Experiment 2, which was preregistered, we extended this logic by testing compliance with a new, specific law introduced by the punishing authority after their initial punishment decision. We predicted that moral agreement with a punished act would reduce subsequent willingness to comply with this new law. In Experiment 3, we used a 2 × 2 factorial design, manipulating moral agreement with the perpetrator’s behavior and punisher’s procedural justice reputation to compare their effects on broad compliance. We predicted that punishing an act participants morally agreed with would reduce broad compliance, that this effect would be larger than any effect of procedural justice reputation, and that it would hold at both high and low levels of procedural justice (i.e., no interaction). In Experiment 4, which was preregistered, we replicated Experiment 3 in a sample of individuals with felonies and misdemeanors on their records to determine whether the observed effects hold in a criminalized population. In Experiment 5, we explored a potential boundary condition by manipulating moral agreement of a minor moral violation for which participants were unlikely to hold strong moral attitudes while controlling for potential confounds in the prior experiments. In all experiments, we tested our hypothesized path model to examine whether moral agreement predicts compliance indirectly through perceptions of punisher legitimacy.

For Experiments 1 to 3, we recruited participants via CloudResearch and compensated them with $0.25 after they completed a Qualtrics questionnaire administered through Amazon’s Mechanical Turk (MTurk). CloudResearch uses enhanced vetting procedures to ensure high-quality data by blocking duplicate IP addresses, suspicious geolocation activity, and known bots. Recent research demonstrates that this sampling strategy yields significantly higher data quality compared to standard MTurk samples (Hauser et al., 2023). We also used CloudResearch’s prescreening feature to ensure that participants who completed the experiment did not previously participate in similar studies from our lab. For Experiment 4, we recruited participants from Connect and paid them $0.50. For Experiment 5, we recruited participants from both CloudResearch and Connect to ensure a sufficiently large sample, compensating CloudResearch participants $0.25 and Connect participants $0.50. We observed no differences in results between the two sources. Our university’s Institutional Review Board approved our research, and we obtained informed consent from all participants prior to their participation. All primary data, processed data, syntax code, and preregistrations are available at https://osf.io/2m4vf/?view_only=851926aa1f2c4dffad6439857c26a7fb

Data were analyzed using SPSS version 29.0 and R version 4.1.1. The design and analysis of Experiments 1, 2, and 4 were preregistered.

## Experiment 1

Experiment 1 was a preregistered experiment that examined our hypothesis that if participants are exposed to punishment of an act that they morally agree with, then they would be less willing to obey the law more broadly, and that this relationship would be mediated by reduced perceptions of punisher legitimacy.

### Method

#### Participants

We recruited 528 participants (224 males, 295 females, 4 nonbinary, and 5 missing). The sample was 75% White, 9% Black, 6% Asian, 6% Hispanic/Latino, and less than 1% Native American. About 3% of participants selected “other” and less than 1% did not identify their race. They ranged in age from 18 to 81 years old (*M* = 44.01, *SD* = 12.67). A sensitivity analysis in G*power (Faul et al., 2007) indicated that our sample size would allow us to reliably detect an effect size of η²*p* = .014 or greater with 80% power and alpha at .05 for a between-subjects analysis of variance (ANOVA).

#### Materials and Procedure

We told participants that they would be reading about a scenario and then we randomly assigned them to one of two moral agreement conditions. In both conditions, participants read about a man named John who was penalized by the local police department for protesting. We manipulated participants’ moral agreement with the man’s action. In the moral agreement condition, participants (*N* = 263) read the following vignette:

John was recently caught violating a public conduct law by engaging in disruptive behavior. He was peacefully protesting against government corruption in a public park, using a megaphone to criticize unethical policies. Most people at the public park supported his cause. After reviewing the case, the local police determined that John deserved a severe penalty of a $1000 fine and 100 hours of community service.

In the moral disagreement condition, participants (N = 265) read the following vignette:

John was recently caught violating a public conduct law by engaging in disruptive behavior. He was peacefully protesting against laws banning animal cruelty in a public park, using a megaphone to argue that people should have the right to treat their animals however they see fit. Most people at the public park were against his cause. After reviewing the case, the local police determined that John deserved a severe penalty of a $1000 fine and 100 hours of community service.

After exposure to condition, we asked all participants to evaluate how morally wrong they perceived John’s actions to be, how much they supported the punishment against John, and how willing they were to do the same action on 7-point Likert-type scales ranging from “not at all” to “completely.” These items acted as manipulation checks to show that our manipulation of moral agreement was successful.

Our primary dependent variables focused on perceptions of punisher legitimacy and broader compliance with the punishing authority following punishment of John’s actions. For legitimacy, participants answered questions about the police department in the scenario. They were asked “How legitimate of an authority do you think the local police department is in the scenario?,” “How much do you trust the local police department in the scenario?,” and “How fair and honest do you think the local police department is in the scenario?.” Given the high reliability of these items (*a* = .89), we averaged across them to form a composite for punisher legitimacy. For broad compliance, participants were asked to imagine that the local police department in the scenario was their own and asked, “If the local police department in the scenario was your own, to what extent would you comply with their orders?” and “If you were penalized by the local police department from the scenario, to what extent would you improve your behavior (e.g., strongly avoid breaking the law again)?.” Given the reliability of these items (*a* = .77), we also averaged across them to create a composite of broad compliance. We measured and analyzed action willingness and broad compliance separately in all studies, which deviates from our preregistration. We did this because we believed that our measure of participants’ willingness to repeat the specific act is closely tied to moral agreement, and therefore would serve better as a manipulation check, whereas our broader compliance measure captures wider intentions to comply with the authority in the future. The latter construct is what we were most interested in.

After participants completed the dependent measures, they completed demographic items and were then fully debriefed and thanked for their participation.

#### Results

First, we computed a series of between-subjects ANOVAs to test whether our moral agreement manipulation successfully affected our manipulation variables. The analysis revealed a significant effect of condition on all three measures: participants in the moral agreement condition viewed the action as less morally wrong *F* (1, 526) = 318.50, *p* < .001; η²*p* = .38, 90% confidence interval [CI] = [.33, .42], were less supportive of punishment against the action *F* (1, 523) = 128.34, *p* < .001; η²*p* = .20, 90% CI = [.15, .25], and reported being more willing to engage in the same behavior themselves *F* (1, 523) = 20.90, *p* < .001; η²*p* = .04, 90% CI = [.02, .07], compared to participants in the moral disagreement condition (Table 1).

**Table 1.** Results for Manipulation Check Variables by Moral Agreement Condition From Experiment 1.

Variable	*p* value	Error *df*	*F*	η²p	Moral agreement *M* (*SE*)	Moral disagreement *M* (*SE*)
Act wrongness	<.001	526	318.50	.38	2.03 (.10)	4.56 (.10)
Punishment support	<.001	523	128.34	.20	2.25 (.12)	4.12 (.12)
Action willingness	<.001	523	20.90	.04	2.76 (.11)	2.08 (.11)

Next, we examined our primary hypotheses that moral agreement with the punished behavior would diminish the legitimacy of the punishing authority and future compliance with that authority (Figure 1). A between-subjects ANOVA revealed a large and significant effect of condition on our composite measure of legitimacy, *F* (1, 524) = 83.82, *p* < .001; η²*p* = .14, 90% CI = [.09, .18], such that participants in the moral agreement condition viewed the police as significantly less legitimate (*M* = 3.15, *SE* = .10) than participants in the moral disagreement condition (*M* = 4.50, *SE* = .10). For our composite measure of broad compliance, we also found a significant effect of condition, *F* (1, 523) = 26.94, *p* < .001; η²*p* = .05, 90% CI = [.02, .08]. Participants in the moral agreement condition reported being significantly less willing to comply with orders and improve their behavior if punished in the future (*M* = 4.78, *SE* = .10) than participants in the moral disagreement condition (*M* = 5.49, *SE* = .10).

**Figure 1.**
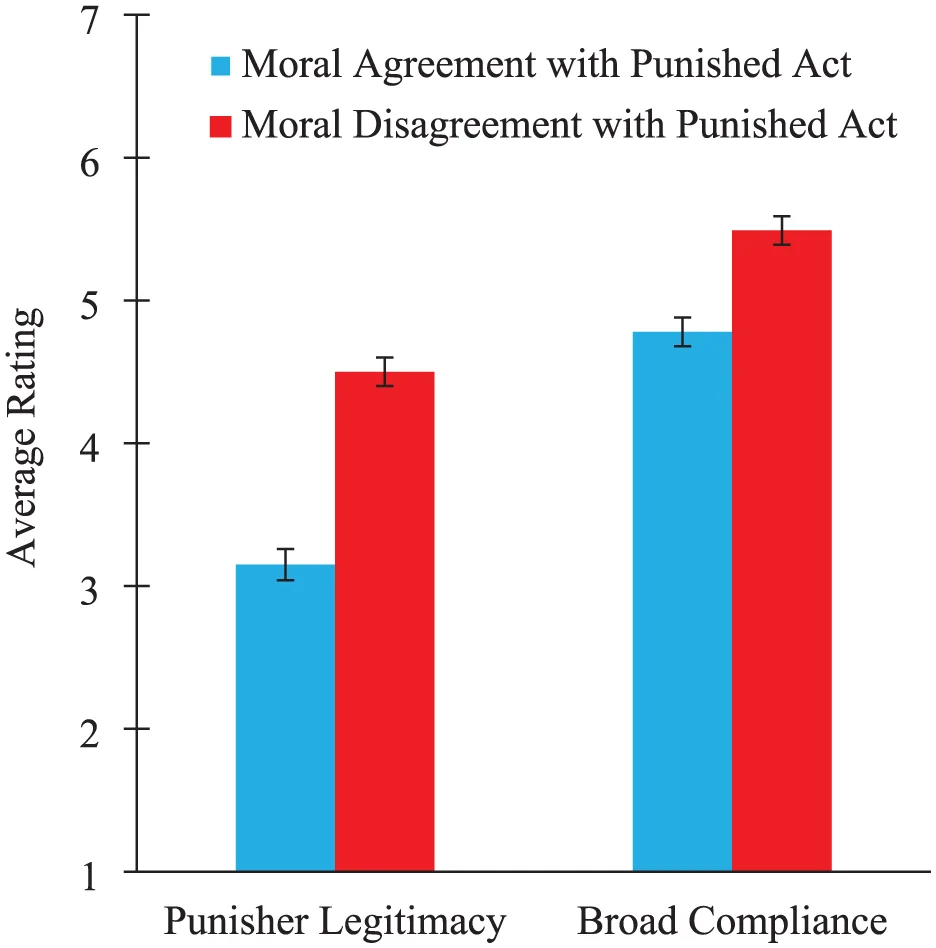
Punisher Legitimacy and Broad Compliance by Moral Agreement Conditions From Experiment 1. Error bars represent standard errors.

Next, we tested the indirect effect of moral agreement condition on broad compliance through perceptions of punisher legitimacy using Model 4 of PROCESS (version 4.0) in SPSS, with 5,000 bias-corrected bootstrap resamples (Hayes, 2017). While our mediation models are based on cross-sectional data, we designed them to support causal interpretation and based them on prior research and theory. For all models, we always experimentally manipulate the first path (moral agreement → legitimacy), and we strongly ground the second path (legitimacy → broad compliance) in prior empirical research linking legitimacy to legal compliance (see Walters & Bolger, 2019, for a meta-analysis; also see Bullock & Green, 2021, for discussion of mediation design considerations). For this, and all mediation models, BCa CI refers to bias-corrected and accelerated confidence intervals. The lettered labels (e.g., *a*, *b*, *ab*) indicate the specific path coefficients estimated within the mediation model. The results corroborated our model (see Figure 2), showing that the moral agreement condition predicted attenuated perceptions of punisher legitimacy (*a* = −1.25, *Bca CI* [−1.52, −.99], *p* < .001) and legitimacy perceptions predicted future compliance intentions and learning from punishment by the police (*b* = .56, *Bca CI* [.48, .63], *p* < .001). The indirect effect of moral agreement condition on compliance through punisher legitimacy was significant (*ab* = −.70, *Bca CI* [−.87, −.54]) and fully mediated the relationship as the direct effect of condition became nonsignificant (*p* = .90) when accounting for legitimacy.

**Figure 2.**
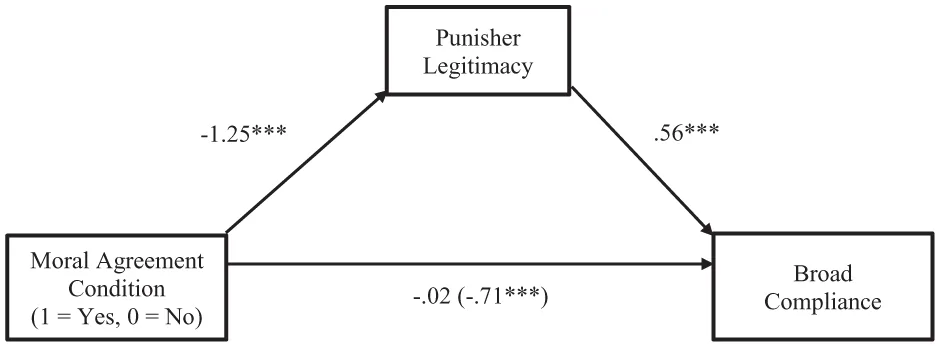
Effect of Moral Agreement Condition on Broad Compliance Through Punisher Legitimacy From Experiment 1. ***p < .001.

## Experiment 2

Experiment 1 demonstrated that when morally agreeable acts were punished, participants perceived the police as less legitimate and reported less willingness to comply and learn from penalties from the police department in the future. We also found support for our theorized mediation model—moral agreement ➔ legitimacy ➔ broad compliance. One concern with our results, however, is that participants may have been assuming different kinds of future laws and punishments depending on whether they agreed or disagreed with the initial punished act, and therefore their reported willingness to comply with the punishing authority may have reflected compliance with different imagined laws across conditions. To account for this possibility, in Experiment 2, we conducted a preregistered extension of Experiment 1 in which we held constant the content of the law by introducing a new, identical rule across conditions. This allowed us to test whether moral agreement with the initial punished act would shape willingness to comply with the new, specific law.

### Method

#### Participants

We recruited 414 participants (187 males, 217 females, 5 nonbinary, and 5 missing). The sample was 74% White, 9% Black, 9% Asian, 5% Hispanic/Latino, and 1% Native American. About 2% of participants selected “other” or did not identify their race. They ranged in age from 19 to 79 years old (*M* = 43.91, *SD* = 12.34). A sensitivity analysis in G*power (Faul et al., 2007) indicated that our sample size would allow us to reliably detect an effect size of η²*p* = .019 or greater with 80% power and alpha at .05 for a between-subjects ANOVA.

#### Materials and Procedure

The materials and procedure were nearly identical to Experiment 1. The only change we made was how we measured our compliance variable. Before responding to our compliance items, participants were told the following:

Imagine that the local police department from the scenario started enforcing a new rule: Beginning next month, the local police department will enforce park closing hours from 8:00 pm to 8:00 am. Being in the park during closing hours will lead to a $100 citation.

They were then asked the same compliance questions used in Experiment 1 but modified to fit the new scenario: (1) “If the local police department in the scenario was your own, how willing would you be to comply with their closing hours rule?” and (2) “If you received a citation from the local police department from the scenario for breaking their closing hours rule, how likely would you be to improve your behavior (e.g., strongly avoid breaking the rule again)?.” We averaged responses across these items to create a composite measure of specific compliance (*a* = .85).

After participants completed the dependent measures, they completed demographic items and were then fully debriefed and thanked for their participation.

### Results

First, we computed a series of between-subjects ANOVAs to test whether our moral agreement manipulation successfully affected our manipulation variables. Like in Experiment 1, the analysis revealed a significant effect of condition on all three measures: participants in the moral agreement condition viewed the action as less morally wrong *F* (1, 408) = 161.55, *p* < .001; η²*p* = .28, 90% CI = [.22, .34], were less supportive of punishment against the action *F* (1, 406) = 68.71, *p* < .001; η²*p* = .15, 90% CI = [.10, .20], and reported being more willing to engage in the same behavior themselves *F* (1, 408) = 25.77, *p* < .001; η²*p* = .06, 90% CI = [.03, .10], compared to participants in the moral disagreement condition (Table 2).

**Table 2.** Results for Manipulation Check Variables by Moral Agreement Condition From Experiment 2.

Variable	*p* value	Error *df*	*F*	η²p	Moral agreement *M* (*SE*)	Moral disagreement *M* (*SE*)
Act wrongness	<.001	408	161.55	.28	2.15 (.12)	4.26 (.12)
Punishment support	<.001	406	68.71	.15	2.27 (.14)	3.84 (.13)
Action willingness	<.001	408	25.77	.06	2.97 (.12)	2.10 (.12)

Next, we examined our primary hypotheses that moral agreement with the punished behavior would diminish the legitimacy of the punishing authority and compliance with the new, specific law introduced by that authority. A between-subjects ANOVA revealed a large and significant effect of condition on our composite measure of legitimacy (Figure 3), *F* (1, 408) = 79.80, *p* < .001; η²*p* = .16, 90% CI = [.11, .22], such that participants in the moral agreement condition viewed the police as significantly less legitimate (*M* = 2.95, *SE* = .11) than participants in the moral disagreement condition (*M* = 4.35, *SE* = .11). For our composite measure of specific compliance, we also found a significant effect of condition, *F* (1, 408) = 9.43, *p* = .002; η²*p* = .02, 90% CI = [.004, .05]. Participants in the moral agreement condition reported being significantly less willing to comply with the new law and improve their behavior if punished for breaking the new law (*M* = 5.48, *SE* = .10) than participants in the moral disagreement condition (*M* = 5.93, *SE* = .10).

**Figure 3.**
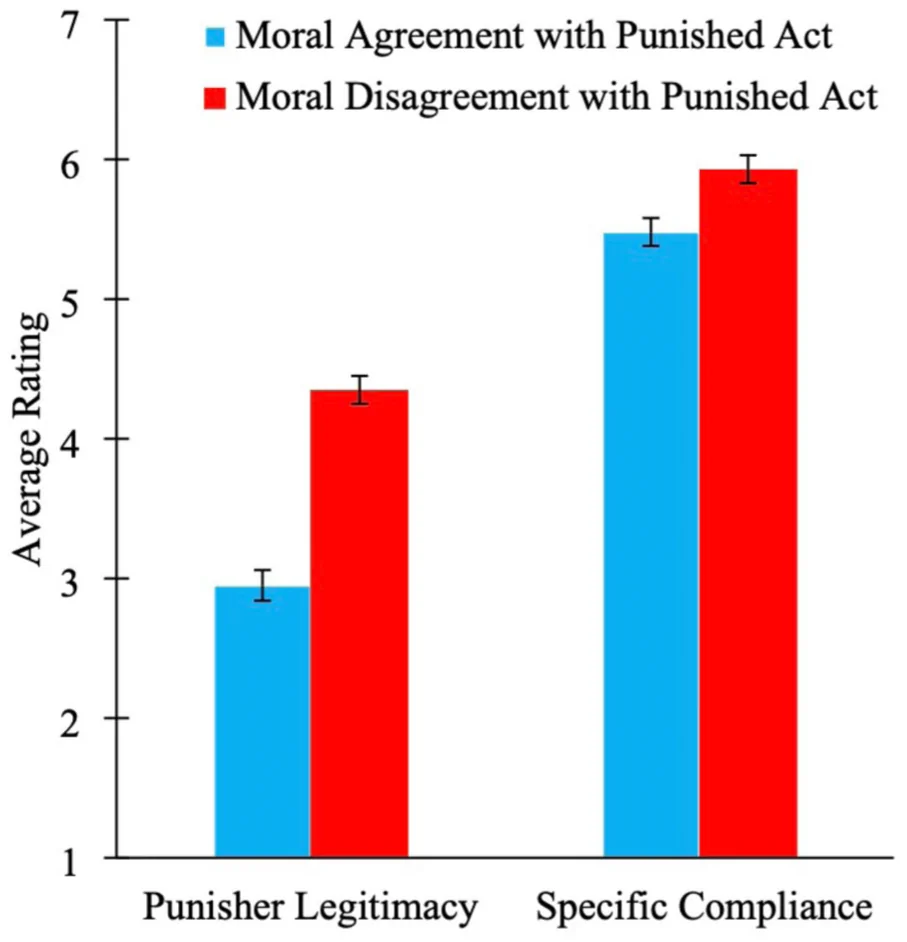
Punisher Legitimacy and Specific Compliance by Moral Agreement Conditions From Experiment 2. Error bars represent standard errors.

Next, we tested the indirect effect of moral agreement condition on specific compliance through perceptions of punisher legitimacy using Model 4 of PROCESS (version 4.0) in SPSS, with 5,000 bias-corrected bootstrap resamples (Hayes, 2017). Results provided further evidence for our theorized model (see Figure 4), showing that the moral agreement condition predicted attenuated perceptions of punisher legitimacy (*a* = −1.40, *Bca CI* [−1.71, −1.09], *p* < .001) and legitimacy perceptions predicted reported compliance with the new law (*b* = .25, *Bca CI* [.16, .34], *p* < .001). The indirect effect of moral agreement condition on specific compliance through punisher legitimacy was significant (*ab* = −.35, *Bca CI* [−.51, −.21]) and fully mediated the relationship as the direct effect of condition became nonsignificant (*p* = .51) when accounting for legitimacy.

**Figure 4.**
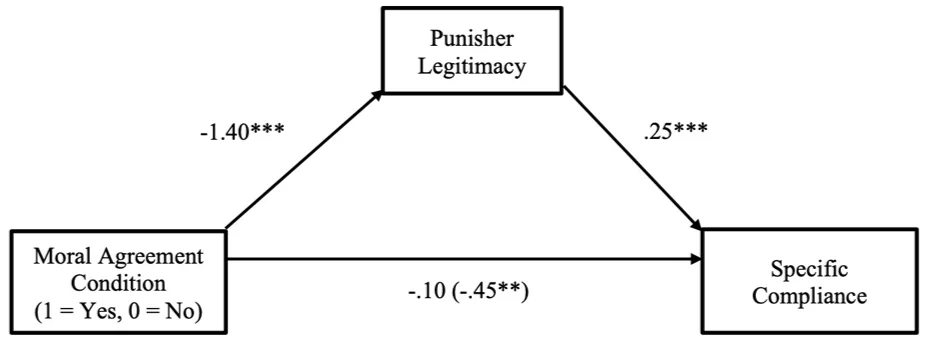
Effect of Moral Agreement Condition on Specific Compliance Through Punisher Legitimacy From Experiment 2. **p < .01. ***p < .001.

## Experiment 3

Experiment 2 replicated the findings of Experiment 1 and extended them by showing that moral agreement with a punished action reduced subsequent compliance even with a newly introduced, specific law. This suggests that our pattern of results for broad compliance with the law is not due to participants simply assuming that they would have to comply with more unjust laws in the future when they morally disagree with laws that authorities enforce in the present. Rather our results suggest that moral disagreement with laws in the present undermines perceptions of authorities’ legitimacy that are crucial to broad compliance in the future.

Notably, in Experiments 1 and 2, participants had no prior information about the legitimacy of the police department. This raises the question of whether a punisher’s procedural justice reputation also contributes independently to their perceived legitimacy and shapes broad compliance, or whether moral agreement remains the primary driver of these effects even when participants have prior reputational information about authorities. To address this, Experiment 3 employed a 2 (agreement vs. disagreement) × 2 (low procedural justice vs. high procedural justice) factorial design. Participants read a vignette about a man penalized by a local police department for a political protest, and we manipulated whether he was punished by a police force with or without a reputation for being procedurally just, and whether the protest was for a morally agreeable or disagreeable cause. We hypothesized that (a) punishing an act that participants morally agreed with would reduce broad compliance relative to punishing a morally disagreeable act, (b) the effect of the moral agreement manipulation on broad compliance would be larger than the effect of the procedural justice reputation manipulation, and (c) the moral agreement effect on broad compliance would hold at both high and low levels of procedural justice (i.e., no interaction).

### Method

#### Participants

We recruited 608 participants (242 males, 354 females, 5 nonbinary, and 7 missing). The sample was 71% White, 11% Black, 6% Asian, 7% Hispanic/Latino, and 1% Native American. About 4% of participants selected “other” or did not identify their race. They ranged in age from 20 to 93 years old (*M* = 42.80, *SD* = 13.07). A sensitivity analysis in G*power (Faul et al., 2007) indicated that our sample size would allow us to reliably detect an effect size of η²*p* = .012 or greater with 80% power and alpha at .05 for a between-subjects ANOVA.

#### Materials and Procedure

We told participants that they would be reading about a scenario, and we randomly assigned them to one of four conditions in a 2 (high vs. low legitimacy) × 2 (moral agreement vs. disagreement) factorial design. In all conditions, participants read about a man named John who was penalized by the local police department for protesting. We manipulated (a) the police department’s reputation for procedural justice—described either as widely respected for its transparency and fairness (high procedural justice) or frequently criticized for corruption and bias (low procedural justice)—and (b) moral agreement with the protest. In the agreement condition, John protested against government corruption, and most people at the public park supported his cause. In the disagreement condition, John protested against laws banning animal cruelty, and most people at the public park opposed his cause. We then told all participants that the police department determined that John deserved a severe penalty of a $1000 fine and 100 hours of community service. See the Supplemental Materials for the full wording of all four vignettes.

Afterward, participants completed the same measures used in Experiments 1 and 2, demographic items, and were then fully debriefed and thanked for their participation. Given that Experiment 2 demonstrated that the effect of moral agreement held even when compliance was measured using a specific law, in Experiment 3, we returned to our broader compliance measure used in Experiment 1, which we also used in the remaining experiments.

### Results

First, we turn our attention to our dependent variables of punisher legitimacy and broad compliance (see Supplementary Tables 1 and 2 for the effects of the conditions on the manipulation check variables). We computed a 2 (moral agreement vs. moral disagreement) × 2 (low procedural justice vs. high procedural justice) between-subjects ANOVA to test how our manipulations affected perceptions of punisher legitimacy (*a* = .88). The analysis revealed a large and significant main effect of moral agreement condition, *F* (1, 600) = 79.57 *p* < .001; η²*p* = .12, 90% CI = [.08, .16], such that participants in the moral agreement condition viewed the police department as significantly less legitimate (*M* = 3.24, *SE* = .09) than participants in the moral disagreement condition (*M* = 4.34, *SE* = .09). There was a smaller but significant main effect of procedural justice condition, *F* (1, 600) = 24.81, *p* < .001; η²*p* = .04, 90% CI = [.02, .07], such that participants in high procedural justice condition viewed the police department as significantly more legitimate (*M* = 4.10, *SE* = .09) than participants in the low procedural justice condition (*M* = 3.48, *SE* = .09). There was no significant interaction between the conditions on legitimacy perceptions, *F* (1, 600) = 1.61, *p* = .21; η²*p* = .00.

For our composite measure of broad compliance (*a* = .80), we also found a significant main effect of moral agreement condition, *F* (1, 598) = 16.27, *p* < .001; η²*p* = .03, 90% CI = [.01, .05]. Participants in the moral agreement condition reported being significantly less willing to comply and learn from penalties coming from the local police department (*M* = 4.80, *SE* = .10) than participants in the moral disagreement condition (*M* = 5.34, *SE* = .10). However, there was no main effect of procedural justice condition, *F* (1, 598) = .00, *p* = .95, η²*p* = .00, nor a significant interaction between the conditions, *F* (1, 598) = .40, *p* = .53; η²*p* = .00. Together, these findings demonstrate that moral attitudes about the punished action exerted a substantially stronger influence on legitimacy and compliance intentions than the punisher’s reputation for procedural justice, as shown in Figure 5.

**Figure 5.**
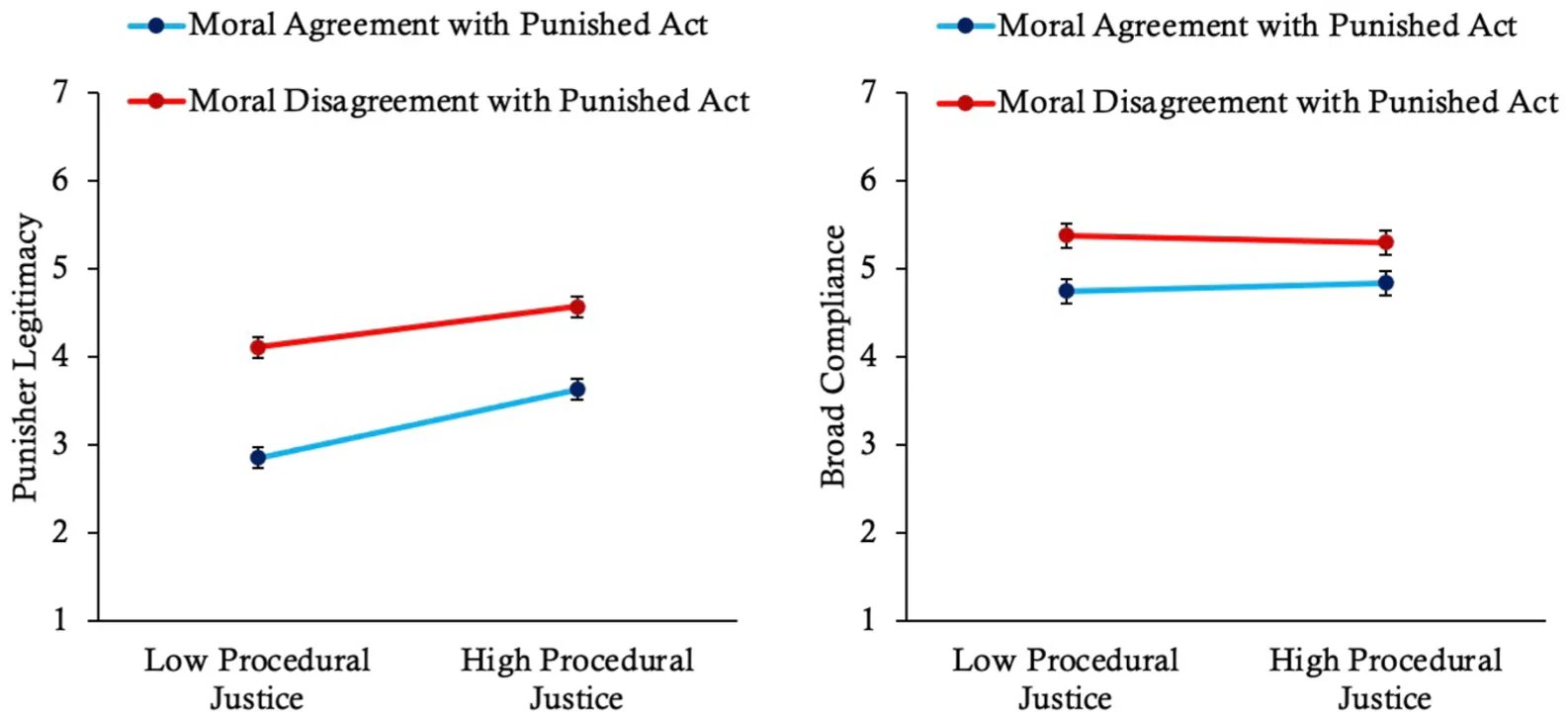
Punisher Legitimacy and Broad Compliance by Moral Agreement and Procedural Justice Conditions From Experiment 3. Error bars represent standard errors.

Given the lack of main effects and interactions with procedural justice reputation on broad compliance, we tested the indirect effect of moral agreement condition (across legitimacy conditions) on compliance through perceptions of punisher legitimacy using Model 4 of PROCESS (version 4.0) in SPSS, with 5,000 bias-corrected bootstrap resamples (Hayes, 2017). Results supported our model (see Figure 6), showing that the moral agreement condition predicted weaker perceptions of police legitimacy (*a* = −1.09, *Bca CI* [−1.34, −.85], *p* < .001) and legitimacy perceptions predicted reported future compliance and learning from punishment by the local police (*b* = .50, *Bca CI* [.42, .57], *p* < .001). The indirect effect of moral agreement condition on compliance through perceptions of punisher legitimacy was significant (*ab* = −.55, *Bca CI* [−.70, −.40]) and fully mediated the relationship as the direct effect of condition became non-significant (*p* = .99) when accounting for legitimacy.

**Figure 6.**
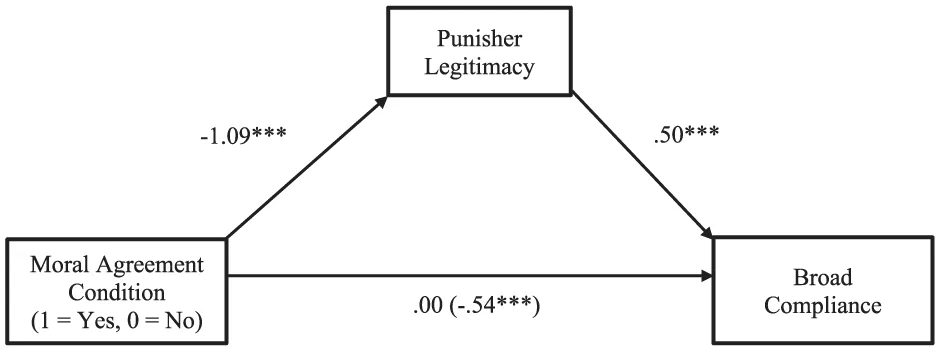
Effect of Moral Agreement Condition on Broad Compliance Through Punisher Legitimacy From Experiment 3. ***p < .001.

## Experiment 4

Experiment 3 demonstrated that moral concerns were a greater predictor of broad compliance than punisher legitimacy under conditions in which participants have information about both. However, while these effects are informative for examining compliance among the broader population, it is unclear whether these effects would generalize to criminalized populations. This is important because it is possible that criminalized populations may be especially suspicious of the legitimacy of punitive authorities (Smoyer et al., 2015), and so they may be affected by legitimacy information more so than moral agreement compared to the broader population (McCarthy & Brunton-Smith, 2018). Understanding how moral beliefs and legitimacy interact to affect broad compliance among criminalized populations is critical because they are the target population that is most affected by punitive policies. To address this concern, in Experiment 4, we preregistered a replication of these results in a sample of individuals with a criminal history.

### Method

#### Participants

We used Connect’s prescreening feature to recruit 466 participants with a felony or misdemeanor on their record. We excluded 194 survey respondents who indicated that they did not have a felony or misdemeanor on their record leaving us with a final sample of 272 participants (176 males, 94 females, and 2 nonbinary). The sample was 79% White, 9% Black, 2% Asian, 5% Hispanic/Latino, and less than 1% Native American. About 4% of participants selected “other” or did not identify their race. They ranged in age from 22 to 79 years old (*M* = 43.61, *SD* = 10.72). A sensitivity analysis in G*power (Faul et al., 2007) indicated that our sample size would allow us to reliably detect an effect size of η²*p* = .03 or greater with 80% power and alpha at .05 for a between-subjects ANOVA.

#### Materials and Procedure

The materials and procedure were exactly identical to Experiment 2.

### Results

Again, we focus our attention to our primary dependent variables of punisher legitimacy and compliance (see Supplementary Tables 3 and 4 for the effects of the conditions on our manipulation check variables). We computed a 2 (moral agreement vs. moral disagreement) × 2 (low procedural justice vs. high procedural justice) between-subjects ANOVA to test how our manipulations affected perceptions of police legitimacy (*a* = .89). The analysis revealed a large and significant main effect of moral agreement condition, *F* (1, 268) = 40.52, *p* < .001; η²*p* = .13, 90% CI = [.07, .19], such that participants in the moral agreement condition viewed the police department as significantly less legitimate (*M* = 2.81, *SE* = .13) than participants in the moral disagreement condition (*M* = 4.00, *SE* = .13). There was a medium and significant main effect of procedural justice condition, *F* (1, 268) = 18.51, *p* < .001; η²*p* = .07, 90% CI = [.02, .12], such that participants in high procedural justice condition viewed the police department as significantly more legitimate (*M* = 3.80, *SE* = .13) than participants in the low procedural justice condition (*M* = 3.00, *SE* = .13). There was no significant interaction between the conditions on police legitimacy perceptions, *F* (1, 268) = 1.14, *p* = .29; η²*p* = .00.

For our composite measure of broad compliance (*a* = .78), we also found a significant and medium main effect of moral agreement condition, *F* (1, 268) = 19.99, *p* < .001; η²*p* = .07, 90% CI = [.02, .12]. Participants in the moral agreement condition reported being significantly less willing to comply and learn from penalties coming from the local police department (*M* = 4.31, *SE* = .15) than participants in the moral disagreement condition (*M* = 5.24, *SE* = .14). There was no main effect of procedural justice condition, *F* (1, 268) = 2.04, *p* = .15, η²*p* = .01, nor a significant interaction between the conditions, *F* (1, 268) = .27, *p* = .61; η²*p* = .00. Replicating Experiment 3, these findings demonstrate the dominant effect of moral attitudes on legitimacy and compliance intentions relative to the punisher’s reputation for procedural justice, as illustrated again in Figure 7.

**Figure 7:**
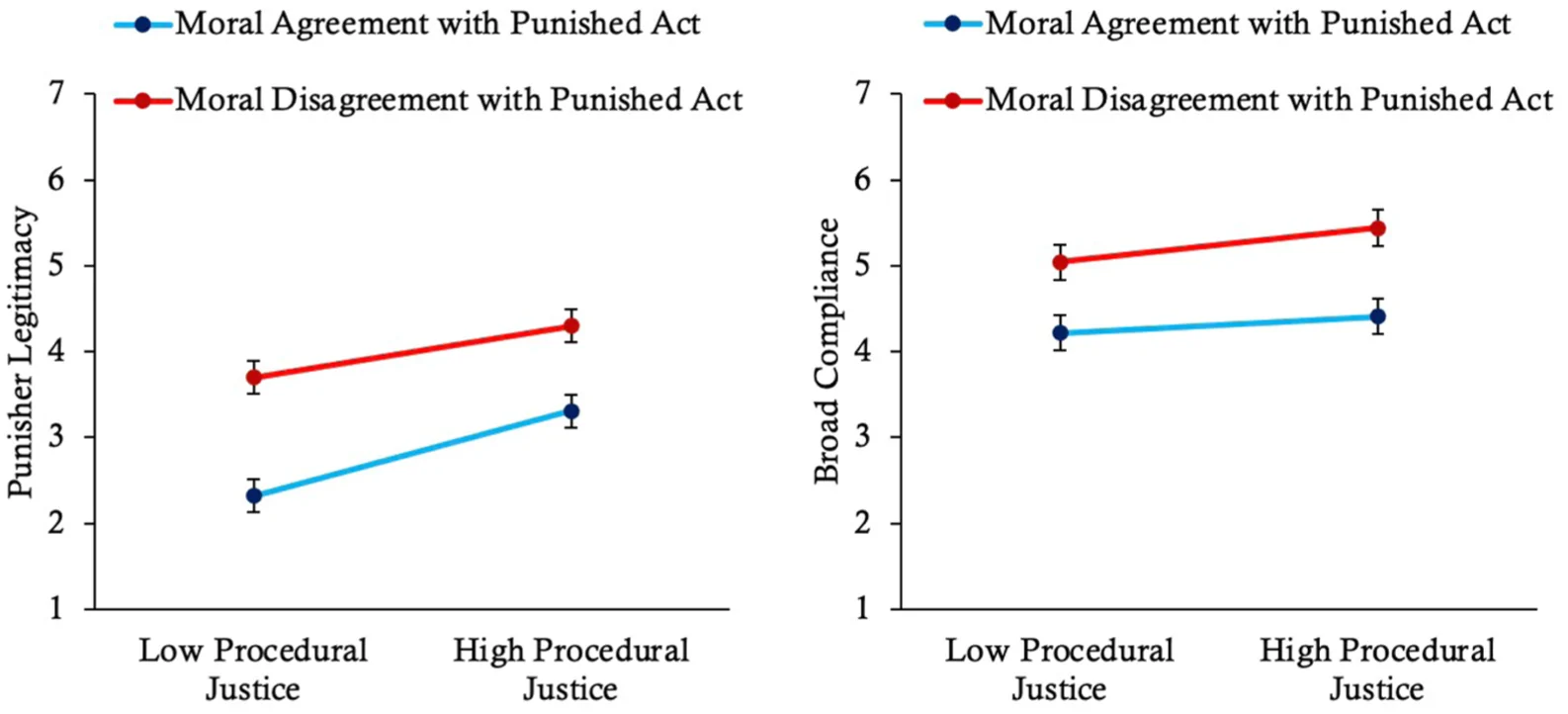
Punisher Legitimacy and Broad Compliance by Moral Agreement and Procedural Justice Conditions From Experiment 4 Error bars represent standard errors.

Given the same pattern of results (i.e., lack of significant effect of the procedural justice manipulation on broad compliance), we ran the same mediation model used in Experiment 3 (Figure 8). Results revealed that the moral agreement condition predicted weaker perceptions of police legitimacy (*a* = −1.20, *Bca CI* [−1.57, −.82], *p* < .001) and legitimacy perceptions predicted reported future compliance intentions (*b* = .52, *Bca CI* [.41, .64], *p* < .001). The indirect effect of moral agreement condition on broad compliance through perceptions of police legitimacy was significant (*ab* = −.63, *Bca CI* [−.87, −.41]) and fully mediated the relationship as the direct effect of moral agreement condition became nonsignificant (*p* = .12) when accounting for legitimacy.

**Figure 8.**
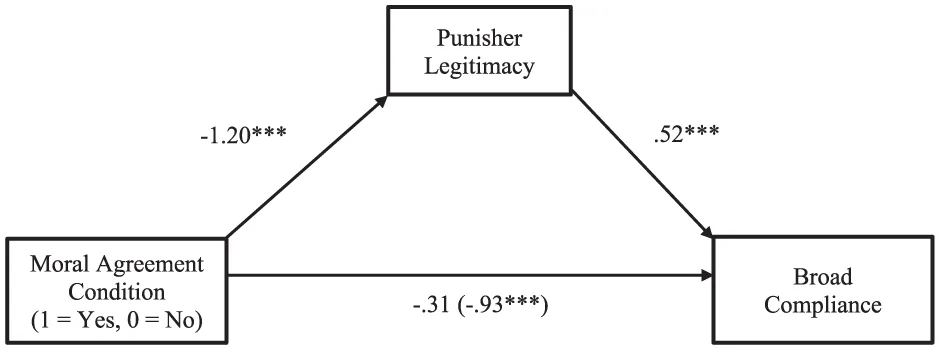
Effect of Moral Agreement Condition on Broad Compliance Through Punisher Legitimacy From Experiment 4. ***p < .001.

## Experiment 5

Experiment 4 replicated the findings of Experiment 3 in a sample with a criminal history. In Experiment 5, we accounted for limitations of the prior experiments. First, the moral agreement condition involved protesting government corruption, which may have inadvertently influenced perceptions of the police themselves, making it less clear whether the effects of moral agreement on perceived legitimacy were due to participants’ agreement with the protest or to the vignette’s implication that the broader government, including the local police, was untrustworthy. Second, the vignettes explicitly stated whether the public supported or opposed the act. We did this to strengthen the moral agreement and disagreement manipulations, but it may have risked conflating participants’ personal moral agreement with perceptions of social norms. Third, although we found that moral agreement consistently predicted broader compliance with the punishing authority more strongly than the punisher’s procedural justice reputation, it remains possible that these effects reflect greater strength of our moral agreement manipulations rather than inherent advantages of moral beliefs over procedural justice in influencing perceptions of legitimacy and broad compliance.

Addressing the aforementioned limitations also allowed us to probe a potential boundary condition of moral agreement. Prior work on the boundary conditions of procedural justice has largely examined cases of strong moral convictions (e.g., abortion attitudes; Bauman & Skitka, 2009; Skitka et al., 2009; Skitka & Houston, 2001; Skitka & Mullen, 2002; Mullen & Skitka, 2006). We asked whether the same pattern holds when moral preferences are weaker. One expectation is that as prior beliefs about a behavior’s moral status become less certain, cues about the punisher’s reputation should play a larger role. This expectation aligns with Bayesian inverse-planning accounts of punishment (Radkani et al., 2025), which propose that observers jointly infer both the wrongness of an act and the punisher’s motives from punitive choices, with the impact of those inferences depending on observers’ priors. Under this view, punishment is more informative to individuals when their moral priors are weak and when they perceive the punishing authority as legitimate, suggesting a tradeoff in which the authority’s reputation for procedural justice becomes more influential as moral priors weaken. Alternatively, people may inherently privilege moral agreement over procedural justice cues, such that even when moral preferences are weaker, agreement with the punished act continues to dominate legitimacy perceptions and compliance intentions.

To address these issues and explore the potential boundary condition, Experiment 5 used a refined 2 (moral agreement vs. disagreement) × 2 (high procedural justice vs. low procedural justice) factorial design in which participants read about the same behavior of distributing fliers in a public park without a permit, but we manipulated whether the fliers promoted antilittering behavior in the park (moral agreement) or argued that people should be free to litter (moral disagreement). This manipulation of moral agreement removes any explicit references to government misconduct or social norm information, allowing us to better isolate moral views from other influences. Moreover, we designed it to be less extreme than the issues used in the previous experiments to achieve a closer match in strength to the procedural justice manipulation (see Supplemental Materials for moral agreement manipulation comparisons between Experiments 3 and 5).

### Method

#### Participants

We used CloudResearch and Connect to recruit 517 participants (256 males, 244 females, 4 nonbinary, and 13 missing). The sample was 69% White, 10% Black, 8% Asian, 7% Hispanic/Latino, and 2% Native American. About 4% of participants selected “other” or did not identify their race. They ranged in age from 18 to 82 years old (*M* = 39.72, *SD* = 11.95). A sensitivity analysis in G*power (Faul et al., 2007) indicated that our sample size would allow us to reliably detect an effect size of η²*p* = .02 or greater with 80% power and alpha at .05 for a between-subjects ANOVA.

#### Materials and Procedure

We told participants that they would be reading about a scenario, and we randomly assigned them to one of four conditions in a 2 (high procedural justice vs. low procedural justice) × 2 (moral agreement vs. moral disagreement) factorial design. In all conditions, participants read about a man named John who was penalized by the local police department for distributing fliers in a public park without proper permits. We manipulated (a) the procedural justice reputation of the police department in the same way we did in the previous experiments, and (b) moral agreement with the flier’s content. In the moral agreement condition, John’s fliers discouraged people from littering in the park. In the moral disagreement condition, his fliers argued that people should be free to litter. We then told all participants that the police determined John deserved a severe penalty of a $500 fine and 10 hours of community service. See the Supplemental Materials for the full wording of all four vignettes.

Afterward, participants completed the same measures used in Experiment 1. We also revised the compliance items to more clearly reflect broader compliance with punishing authorities (i.e., “If the local police department in the scenario was your own, how willing would you be to comply with their orders in general?” and “If you were penalized by the police department from the scenario for breaking any law, how likely would you be to improve your behavior in general [e.g., strongly avoid breaking any law again]?”). The survey concluded with demographic questions, after which we debriefed and thanked participants for their participation.

### Results

We focus on our primary dependent variables of punisher legitimacy and broad compliance (see Supplementary Tables 5 and 6 for the effects of the conditions on our manipulation check variables). We computed a 2 (moral agreement vs. moral disagreement) × 2 (low procedural justice vs. high procedural justice) between-subjects ANOVA to test how our new manipulations affected perceptions of police legitimacy (*a* = .89). The analysis revealed a large and significant main effect of moral agreement condition, *F* (1, 503) = 64.43, *p* < .001; η²*p* = .11, 90% CI = [.07, .16], such that participants in the moral agreement condition viewed the police department as significantly less legitimate (*M* = 3.36, *SE* = .10) than participants in the moral disagreement condition (*M* = 4.50, *SE* = .10). There was also a significant main effect of procedural justice condition, *F* (1, 503) = 25.71, *p* < .001; η²*p* = .05, 90% CI = [.02, .08], such that participants in high procedural justice condition viewed the police department as significantly more legitimate (*M* = 4.29, *SE* = .10) than participants in the low procedural justice condition (*M* = 3.57, *SE* = .10). There was no significant interaction between procedural justice and moral agreement conditions, *F* (1, 503) = .45, *p* = .50; η²*p* = .00.

For our composite measure of broad compliance (*a* = .85), we also found a significant main effect of moral agreement condition, *F* (1, 502) = 13.01, *p* < .001; η²*p* = .03, 90% CI = [.01, .05]. Participants in the moral agreement condition reported being significantly less willing to comply and learn from penalties coming from the local police department (*M* = 4.60, *SE* = .10) than participants in the moral disagreement condition (*M* = 5.12, *SE* = .10). There was no main effect of procedural justice condition, *F* (1, 502) = 2.77, *p* = .10, η²*p* = .01, nor a significant interaction between conditions on compliance, *F* (1, 502) = 1.43, *p* = .23; η²*p* = .00. Replicating and extending Experiments 3 and 4, these findings show that moral agreement exerted a stronger influence on punisher legitimacy and compliance intentions than the punisher’s procedural justice reputation, even under a weaker moral attitude manipulation (see Figure 9).

**Figure 9.**
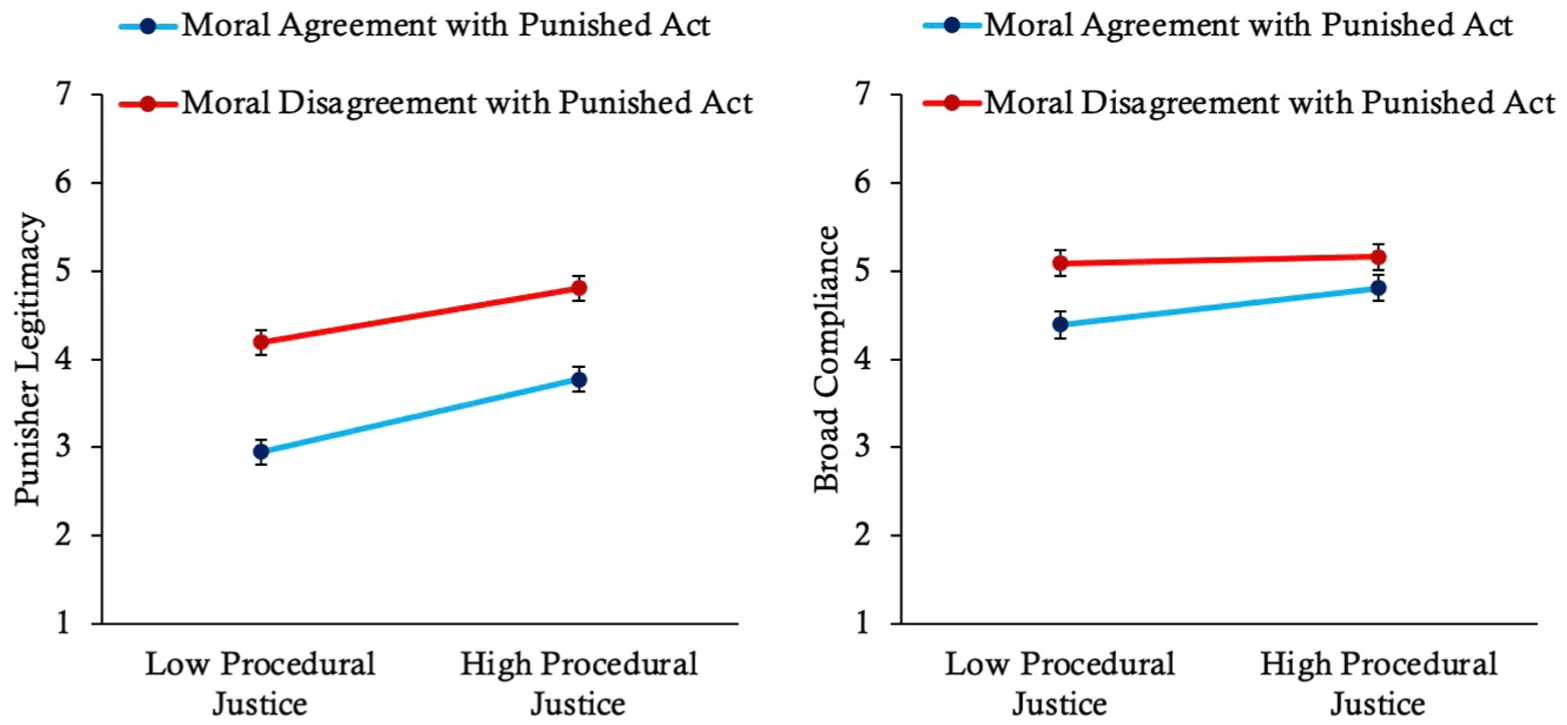
Punisher Legitimacy and Broad Compliance by Moral Agreement and Procedural Justice Conditions From Experiment 5. Error bars represent standard errors.

We ran the same mediation model used in Experiments 3 and 4. Results again supported our model (see Figure 10), showing that the moral agreement condition predicted weaker perceptions of police legitimacy (*a* = −1.15, *Bca CI* [−1.43, −.86], *p* < .001), and legitimacy perceptions predicted greater reported future compliance (*b* = .51, *Bca CI* [.44, .59], *p* < .001). The indirect effect of moral agreement condition on broad compliance through perceptions of police legitimacy was significant (*ab* = −.59, *Bca CI* [−.77, −.42]) and fully mediated the relationship as the direct effect of condition became nonsignificant (*p* = .64) when accounting for legitimacy.

**Figure 10.**
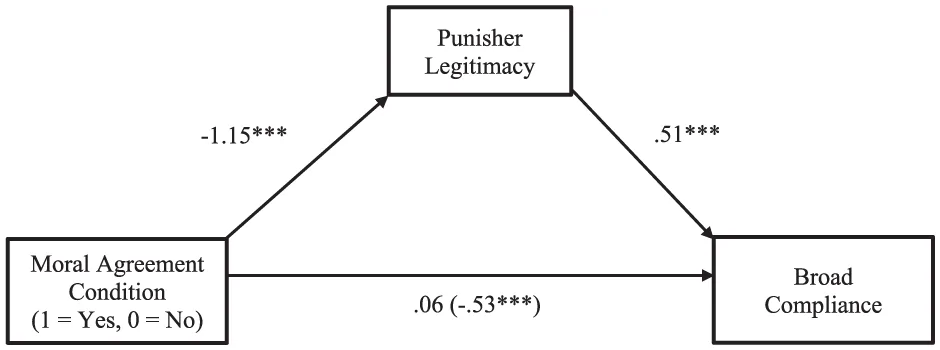
Effect of Moral Agreement Condition on Broad Compliance Through Punisher Legitimacy From Experiment 5. ***p < .001.

## Discussion

The present research demonstrates that moral agreement with criminalized behavior significantly reduces perceptions of punisher legitimacy and weakens broader compliance with punishing authorities. In Experiment 1, participants who morally agreed with a perpetrator’s behavior viewed the punishing authority as less legitimate and reported lower willingness to comply with them in the future. A mediation model supported our theoretical account, showing that the effect of moral agreement on compliance was driven by diminished perceptions of legitimacy. Experiment 2 replicated these effects and ruled out the possibility that they were driven by participants imagining different laws across conditions by showing that moral agreement with a punished action reduced compliance with a newly introduced, specific law. Experiment 3 extended these findings by orthogonally manipulating both moral agreement and punisher legitimacy, revealing that moral agreement was the stronger predictor of compliance, with no significant interaction between the two factors. Experiment 4 replicated these findings in a sample of individuals with a criminal history, reinforcing our results in a population directly affected by punitive policies and at greater risk of future system involvement. Experiment 5 extended our results to show that even in cases wherein participants likely do not hold strong moral opinions, moral beliefs still drive perceptions of legitimacy and broad compliance, suggesting that these effects extend beyond the literature on absolute and nonnegotiable moral convictions (Skitkaetal.,2005).

These findings underscore the need to look beyond procedural justice interventions to moral belief interventions to effectively address criminal behavior (Alam & Rai, 2025b). This is particularly relevant as societies become increasingly pluralistic in their values and moral disagreements within societies deepen (Huo et al., 1996; Jackson & Medvedev, 2024). More specifically, the results imply that punitive policies will have limited effects on compliance when individuals view crimes as morally acceptable and suggest that alternative crime control approaches may need to be pursued by justice institutions. For example, restorative justice interventions focus on engaging offenders in dialogue with victims and their communities, with the goal of reintegrating offenders into the community (Braithwaite, 2002). While some research suggests that the efficacy of restorative justice may stem from providing victims with a sense of voice (Sherman et al., 2005), our findings suggest that—among other potential mechanisms—restorative justice may also be effective by facilitating genuine moral belief change in offenders (Dhami, 2012; Tangney et al., 2014).

Interestingly, our results emerged not only for highly moralized acts such as protesting government corruption, but also for comparatively weakly moralized issues such as distributing fliers about littering. That moral agreement outweighed procedural justice even in this context suggests that agreement with punished acts exerts a robust influence on legitimacy and compliance. This finding also bears on Bayesian inverse-planning models of punishment (Radkani et al., 2025), which predict a tradeoff in which punisher legitimacy cues should become more influential when moral priors about actions are weak. Instead, our data suggest that moral agreement retains privileged weight over procedural justice information even when priors are relatively weak, indicating that procedural justice and moral agreement are not interchangeable sources of information and that procedural justice may matter most when moral preferences are particularly weak or absent.

Our experiments offer several opportunities for future research. First, our compliance measures relied on self-reports rather than behavioral outcomes. Future work should incorporate behavioral methods to examine how moral disagreement and punisher legitimacy interact to influence behavioral compliance with the law. Second, our samples were composed predominantly of White Americans. This may have shaped how participants interpreted the legitimacy of the police as White Americans are less likely to face criminalization and may perceive less risk in challenging legal authority (Kovera, 2019). Moreover, Black Americans and other communities that face greater involvement with law enforcement may enter punitive contexts with elevated distrust toward legal authorities (MacDonald & Stokes, 2006). As a result, even when they morally disagree with a transgression, they may still view punishers as illegitimate. Third, although Experiment 5 narrowed the gap, the moral agreement manipulation still had a stronger effect on action wrongness than the procedural justice manipulation had on perceived legitimacy. This imbalance limits the precision of our comparisons of their effects on compliance. Future work should explore ways to equalize manipulation strength across the constructs and pinpoint the moral preference threshold at which legitimacy information influences broad compliance, thereby testing this boundary condition more cleanly.

## Conclusion

Across five experiments, we find that moral agreement with punished behavior consistently reduces perceptions of a punisher’s legitimacy, which in turn reduces willingness to comply with laws broadly. The effects of moral agreement on perceptions of legitimacy were stronger than manipulations of procedural justice that should be more directly relevant for legitimacy. Our effects are robust across general populations and criminalized individuals, hold even when we measure compliance with respect to a newly introduced, specific law, and persist in contexts where moral agreement is relatively weak. Our results suggest that the perceived morality of punished behavior, and not merely the procedural fairness of the punisher, plays a central role in shaping perceptions of legitimacy and broader compliance with punishing authorities. These findings challenge conventional models that emphasize institutional reputation alone and highlight the importance of accounting for moral beliefs in fostering compliance with the law.

## Supplemental Material

sj-docx-1-psp-10.1177_01461672251385772 – Supplemental material for Moral Agreement With Punished Acts Decreases Perceptions of Punisher Legitimacy and Willingness to Obey the Law Supplemental material, sj-docx-1-psp-10.1177_01461672251385772 for Moral Agreement With Punished Acts Decreases Perceptions of Punisher Legitimacy and Willingness to Obey the Law by Raihan Alam and Tage S. Rai in Personality and Social Psychology Bulletin

## Data Availability

All data are available on Open Science Framework (https://osf.io/2m4vf/?view_only=851926aa1f2c4dffad6439857c26a7fb).
