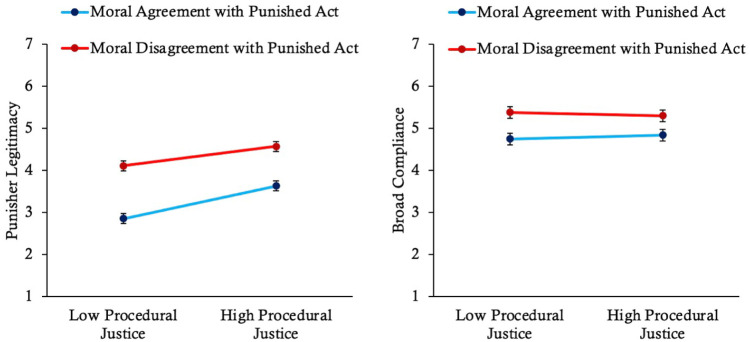# Erratum to “Moral Agreement With Punished Acts Decreases Perceptions of Punisher Legitimacy and Willingness to Obey the Law”

**DOI:** 10.1177/01461672261416841

**Published:** 2026-01-29

Alam, R., & Rai, T. S. (2025). Moral agreement with punished acts decreases perceptions of punisher legitimacy and willingness to obey the law. *Personality and Social Psychology Bulletin*, *0*(0). https://doi.org/10.1177/01461672251385772

The online version of this article has been updated to include the correct version of Figures 3, 4, 5, 7, and 9 below.

**Figure 3.**
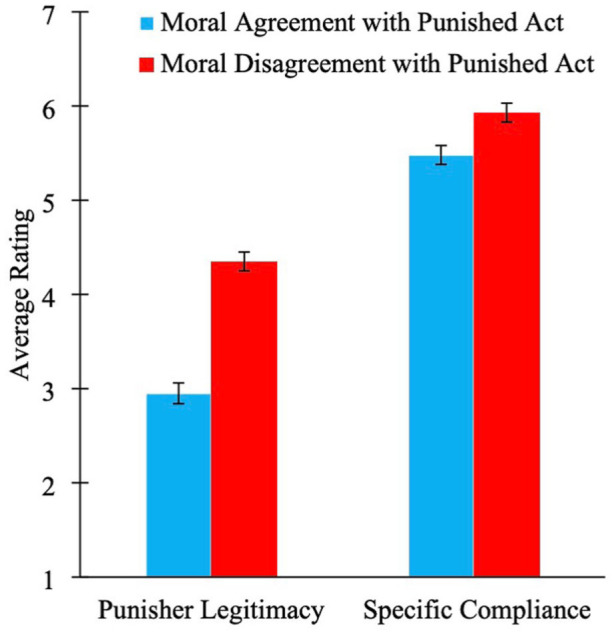


**Figure 4.**
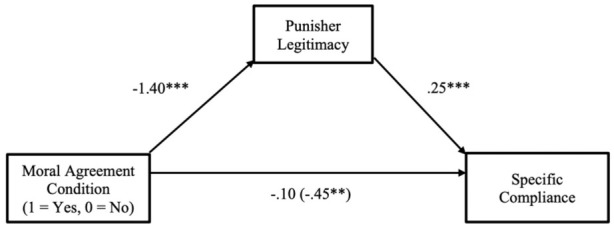


**Figure 5.**
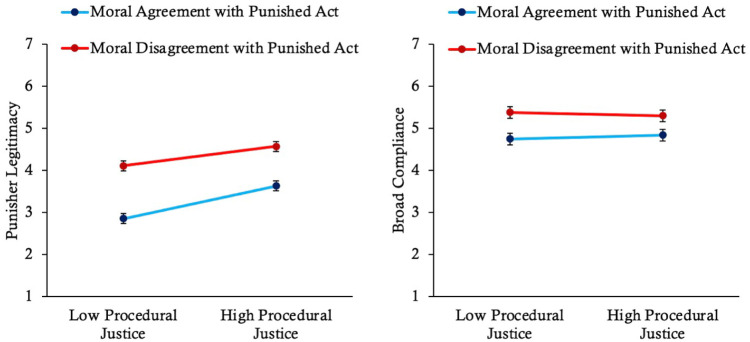


**Figure 7.**
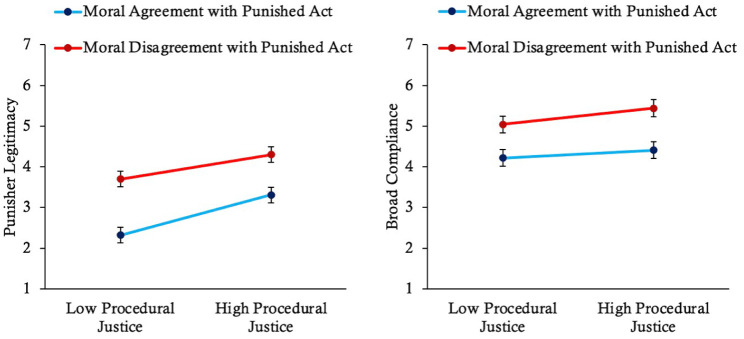


**Figure 9.**