# Supplementary material for: Moral Agreement With Punished Acts Decreases Perceptions of Punisher Legitimacy and Willingness to Obey the Law
Source: Pers Soc Psychol Bull. 2025 Nov 20;52(12):4011–24. doi: 10.1177/01461672251385772 (PMC13736283; doi:10.1177/01461672251385772)
Supplement: Supplementary file 1 [file sj-docx-1-psp-10.1177_01461672251385772.docx]

**Supplemental Information**

**Measures and Manipulations**

**Full Vignettes for Experiments 3 and 4**

**High Legitimacy and Moral Agreement Condition:**

John was recently caught violating a public conduct law by engaging in disruptive behavior. He was peacefully protesting against government corruption in a public park, using a megaphone to criticize unethical policies. Most people at the public park supported his cause. The case was handled by the local police department, which is widely respected in the community for its transparency, fairness, and consistent public approval ratings. After reviewing the case, the local police determined that John deserved a severe penalty of a $1000 fine and 100 hours of community service.

**Low Legitimacy and Moral Agreement Condition:**

John was recently caught violating a public conduct law by engaging in disruptive behavior. He was peacefully protesting against government corruption in a public park, using a megaphone to criticize unethical policies. Most people at the public park supported his cause. The case was handled by the local police department, which is frequently criticized in the community for corruption, bias, and low public approval ratings. After reviewing the case, the local police determined that John deserved a severe penalty of a $1000 fine and 100 hours of community service.

**High Legitimacy and Moral Disagreement Condition:**

John was recently caught violating a public conduct law by engaging in disruptive behavior. He was peacefully protesting against laws banning animal cruelty in a public park, using a megaphone to argue that people should have the right to treat their animals however they see fit. Most people at the public park were against his cause. The case was handled by the local police department, which is widely respected in the community for its transparency, fairness, and consistent public approval ratings. After reviewing the case, the local police determined that John deserved a severe penalty of a $1000 fine and 100 hours of community service.

**Low Legitimacy and Moral Disagreement Condition:**

John was recently caught violating a public conduct law by engaging in disruptive behavior. He was peacefully protesting against laws banning animal cruelty in a public park, using a megaphone to argue that people should have the right to treat their animals however they see fit. Most people at the public park were against his cause. The case was handled by the local police department, which is frequently criticized in the community for corruption, bias, and low public approval ratings. After reviewing the case, the local police determined that John deserved a severe penalty of a $1000 fine and 100 hours of community service.

**Full Vignettes for Experiment 5**

**High Legitimacy and Moral Agreement Condition:**

John was recently caught violating a public conduct law. He had been distributing fliers in a public park without the required permits. The fliers encouraged people to avoid littering and to keep the park clean. The case was handled by the local police department, which is widely respected in the community for its transparency, fairness, and consistent public approval ratings. After reviewing the case, the local police determined that John deserved a severe penalty of a $500 fine and 10 hours of community service.

**Low Legitimacy and Moral Agreement Condition:**

John was recently caught violating a public conduct law. He had been distributing fliers in a public park without the required permits. The fliers encouraged people to avoid littering and to keep the park clean. The case was handled by the local police department, which is frequently criticized in the community for corruption, bias, and low public approval ratings. After reviewing the case, the local police determined that John deserved a severe penalty of a $500 fine and 10 hours of community service.

**High Legitimacy and Moral Disagreement Condition:**

John was recently caught violating a public conduct law. He had been distributing fliers in a public park without the required permits. The fliers argued that people should be free to litter and that rules against it were unnecessary. The case was handled by the local police department, which is widely respected in the community for its transparency, fairness, and consistent public approval ratings. After reviewing the case, the local police determined that John deserved a severe penalty of a $500 fine and 10 hours of community service.

**Low Legitimacy and Moral Disagreement Condition:**

John was recently caught violating a public conduct law. He had been distributing fliers in a public park without the required permits. The fliers argued that people should be free to litter and that rules against it were unnecessary. The case was handled by the local police department, which is frequently criticized in the community for corruption, bias, and low public approval ratings. After reviewing the case, the local police determined that John deserved a severe penalty of a $500 fine and 10 hours of community service.

**Dependent Variables for all Experiments** (all answered from 1, not at all, to 7, completely)

How morally wrong do you think John's action was in the scenario?

How legitimate of an authority do you think the local police department is in the scenario?

How much do you trust the local police department in the scenario?

How fair and honest do you think the local police department is in the scenario?

How supportive are you of the local police department's punishment for John in the scenario?

Given what happened to John, how willing would you be to engage in the same protest behavior?

Imagine that the local police department from the scenario started enforcing a new rule: Beginning next month, the local police department will enforce park closing hours from 8:00 pm to 8:00 am. Being in the park during closing hours will lead to a $100 citation. (version used in Experiment 2)

If the local police department in the scenario was your own, how willing would you be to comply with their closing hours rule? (version used in Experiment 2)

If you received a citation from the local police department from the scenario for breaking their closing hours rule, how likely would you be to improve your behavior (e.g., strongly avoid breaking the rule again)? (version used in Experiment 2)

Imagine that the local police department from the scenario was your own local police department. Please answer the following questions below. (version used in Experiments 3 and 4)

If the local police department in the scenario was your own, to what extent would you comply with their orders? (version used in Experiments 3 and 4)

If you were penalized by the local police department from the scenario, to what extent would you improve your behavior (e.g., strongly avoid breaking the law again)? (version used in Experiments 3 and 4)

If the local police department in the scenario was your own, how willing would you be to comply with their orders in general? (version used in Experiment 5)

If you were penalized by the police department from the scenario for breaking any law, how likely would you be to improve your behavior in general (e.g., strongly avoid breaking any law again)?” (version used in Experiment 5)

How strong and powerful do you think the local police department is in the scenario? (only asked in Experiment 2).

How moral of a person do you think John is in the scenario? (only asked in Experiment 2).

**Demographic Variables**

Here is a 7-point scale on which the political views that people might hold are arranged from extremely liberal to extremely conservative.

- Extremely liberal
- Liberal
- Slightly Liberal
- Moderate: Middle of the Road
- Slightly conservative
- Conservative
- Extremely conservative

Please indicate your age:

__

What is your sex:

- Male
- Female
- Do not identify

How would you describe your class?

- Upper class
- Upper middle class
- Middle class
- Lower middle class
- Working class/lower class

What is your ethnicity?

- African-American/Black
- Asian-American/Asian
- European-American/White
- East Indian
- Hispanic/Chicano/Latino
- Native American
- Other (Please indicate) _______

What is the highest level of education you have completed?

- High school or less
- 2-year college (associate’s degree)
- 4-year college (BA, BS)
- Masters degree (MA, MS)
- PhD, MD, JD, or other higher degree

**Supplementary Analyses**

**Table S1**. Correlation Matrix of Variables from Experiment 1.

|  | Action Wrongness | Punishment Support | Action Willingness | Punisher Legitimacy | Broad  Compliance |
| --- | --- | --- | --- | --- | --- |
| Action Wrongness |  | 0.72*** | -0.26* | 0.64*** | 0.38** |
| Punishment Support |  |  | -0.27* | 0.83*** | 0.48** |
| Action Willingness |  |  |  | -0.29* | -0.53*** |
| Punisher Legitimacy |  |  |  |  | 0.58*** |

**Table S2**. Correlation Matrix of Variables from Experiment 2.

|  | Action Wrongness | Punishment Support | Action Willingness | Punisher Legitimacy | Compliance |
| --- | --- | --- | --- | --- | --- |
| Action Wrongness |  | 0.67*** | -0.21*** | 0.64*** | 0.20** |
| Punishment Support |  |  | -0.19* | 0.83*** | 0.18** |
| Action Willingness |  |  |  | -0.26*** | -0.41*** |
| Punisher Legitimacy |  |  |  |  | 0.30*** |

**Table S3**. Results for Manipulation Check Variables by Moral Agreement Condition from Experiment 3.

| Variable | *p*-value | Error *df* | *F* | η²p | Moral Agreement *M* (*SE*) | Moral Disagreement *M* (*SE*) |
| --- | --- | --- | --- | --- | --- | --- |
| Action Willingness | < .001 | 600 | 40.32 | .06 | 2.83 (.09) | 2.00 (.09) |
| Action Wrongness | < .001 | 604 | 213.07 | .26 | 2.30 (.10) | 4.35 (.10) |
| Punishment Support | < .001 | 600 | 126.3 | .17 | 2.28 (.11) | 4.02 (.11) |

**Table S4**. Results for Manipulation Check Variables by Procedural Justice Condition from

Experiment 3.

| Variable | *p*-value | Error *df* | *F* | η²p | High Procedural Justice  *M* (*SE*) | Low Procedural Justice  *M* (*SE*) |
| --- | --- | --- | --- | --- | --- | --- |
| Action Willingness | .93 | 600 | 0.01 | .00 | 2.42 (.09) | 2.41 (.09) |
| Action Wrongness | .73 | 604 | 0.12 | .00 | 3.33 (.10) | 3.32 (.10) |
| Punishment Support | .09 | 600 | 2.82 | .01 | 2.96 (.16) | 2.52 (.16) |

**Table S5**. Correlation Matrix of Variables from Experiment 3.

|  | Action Wrongness | Punishment Support | Action Willingness | Punisher Legitimacy | Broad  Compliance |
| --- | --- | --- | --- | --- | --- |
| Action Wrongness |  | 0.69*** | -0.27* | 0.57*** | 0.34** |
| Punishment Support |  |  | -0.25* | 0.79*** | 0.41** |
| Action Willingness |  |  |  | -0.31** | -0.56*** |
| Punisher Legitimacy |  |  |  |  | 0.49** |

**Table S6**. Results for Manipulation Check Variables by Moral Agreement Condition from

Experiment 4.

| Variable | *p*-value | Error *df* | *F* | η²p | Moral Agreement *M* (*SE*) | Moral Disagreement *M* (*SE*) |
| --- | --- | --- | --- | --- | --- | --- |
| Action Willingness | < .001 | 267 | 15.23 | .05 | 3.06 (.16) | 2.18 (.16) |
| Action  Wrongness | < .001 | 268 | 90.05 | .25 | 1.90 (.15) | 3.94 (.15) |
| Punishment Support | < .001 | 267 | 53.83 | .17 | 1.92 (.16) | 3.56 (.15) |

**Table S7**. Results for Manipulation Check Variables by Procedural Justice Condition from

Experiment 4.

| Variable | *p*-value | Error *df* | *F* | η²p | High Procedural Justice  *M* (*SE*) | Low Procedural Justice  *M* (*SE*) |
| --- | --- | --- | --- | --- | --- | --- |
| Action Willingness | .17 | 267 | 1.91 | .01 | 2.89 (.16) | 2.35 (.16) |
| Action  Wrongness | .42 | 268 | 0.64 | .00 | 2.91 (.15) | 2.93 (.15) |
| Punishment Support | .047 | 267 | 3.97 | .02 | 2.96 (.16) | 2.52 (.16) |

**Table S8**. Correlation Matrix of Variables from Experiment 4.

|  | Action Wrongness | Punishment Support | Action Willingness | Punisher Legitimacy | Broad  Compliance |
| --- | --- | --- | --- | --- | --- |
| Action Wrongness |  | 0.68*** | -0.29* | 0.58*** | 0.34** |
| Punishment Support |  |  | -0.25* | 0.79*** | 0.43** |
| Action Willingness |  |  |  | -0.30** | -0.52*** |
| Punisher Legitimacy |  |  |  |  | 0.53*** |

**Table S9**. Results for Manipulation Check Variables by Moral Agreement Condition from

Experiment 5.

| Variable | *p*-value | Error *df* | *F* | η²p | Moral Agreement *M* (*SE*) | Moral Disagreement *M* (*SE*) |
| --- | --- | --- | --- | --- | --- | --- |
| Action Willingness | < .001 | 502 | 12.24 | .02 | 2.87 (.11) | 2.33 (.11) |
| Action Wrongness | < .001 | 503 | 91.09 | .15 | 2.53 (.11) | 3.96 (.11) |
| Punishment Support | < .001 | 501 | 113.83 | .19 | 2.51 (.12) | 4.31 (.12) |

**Table S10**. Results for Manipulation Check Variables by Procedural Justice Condition from

Experiment 5.

| Variable | *p*-value | Error *df* | *F* | η²p | High Procedural Justice  *M* (*SE*) | Low Procedural Justice  *M* (*SE*) |
| --- | --- | --- | --- | --- | --- | --- |
| Action Willingness | .12 | 502 | 2.4 | .01 | 2.60 (.11) | 2.59 (.11) |
| Action Wrongness | .048 | 503 | 3.91 | .01 | 3.39 (.11) | 3.10 (.11) |
| Punishment Support | .017 | 501 | 5.78 | .01 | 3.61 (.12) | 3.21 (.12) |

**Table S11**. Correlation Matrix of Variables from Experiment 5.

|  | Action Wrongness | Punishment Support | Action Willingness | Punisher Legitimacy | Broad  Compliance |
| --- | --- | --- | --- | --- | --- |
| Action Wrongness |  | 0.72*** | -0.02 | 0.62*** | 0.28* |
| Punishment Support |  |  | -0.07 | 0.79*** | 0.40** |
| Action Willingness |  |  |  | -0.16 | -0.47** |
| Punisher Legitimacy |  |  |  |  | 0.53*** |

**Analyses of the Moral Agreement Manipulations between Experiments 3 and 5.**

We tested whether participants in the moral disagreement condition from Experiment 5 (John distributing pro-littering fliers) viewed the act as more than “moderately wrong” by comparing wrongness ratings to the 7-point midpoint (4 = moderately wrong). Results from our one-sample test revealed that the difference was non-significant, *t*(253) = -.41, *p* = .69, indicating the act was not treated by participants as a particularly severe moral violation.

We also compared the moral agreement manipulation on act wrongness across experiments using a z-test for the difference between Cohen’s *d*. We found that the effect was smaller in Experiment 5 than Experiment 3 (*d* = .85 vs. 1.19; *z* = 2.67, *p* = .008). Applying the same test to the procedural justice manipulation on perceived punisher legitimacy showed stability across studies (*d* = .43 in Experiment 5 vs. .38 in Experiment 3; *z* = 0.38, *p* = .71). Descriptively, the gap between manipulations narrowed: Experiment 3 mean differences on Δaction wrongness = 2.05 vs. Δlegitimacy = .62; Experiment 5 differences on Δaction wrongness = 1.43 vs. Δlegitimacy = .72.
